# Microscale poroelastic metamodel for efficient mesoscale bone remodelling simulations

**DOI:** 10.1007/s10237-017-0939-x

**Published:** 2017-08-09

**Authors:** C. C. Villette, A. T. M. Phillips

**Affiliations:** Structural Biomechanics, Department of Civil and Environmental Engineering, Imperial College London, London, UK

**Keywords:** Metamodel, Bone remodelling, Microscale, Poroelastic, Mesoscale, Structural

## Abstract

Bone functional tissue adaptation is a multiaspect physiological process driven by interrelated mechanical and biological stimuli which requires the combined activity of osteoclasts and osteoblasts. In previous work, the authors developed a phenomenological mesoscale structural modelling approach capable of predicting internal structure of the femur based on daily activity loading, which relied on the iterative update of the cross-sectional areas of truss and shell elements representative of trabecular and cortical bones, respectively. The objective of this study was to introduce trabecular reorientation in the phenomenological model at limited computational cost. To this aim, a metamodel derived from poroelastic microscale continuum simulations was used to predict the functional adaptation of a simplified proximal structural femur model. Clear smooth trabecular tracts are predicted to form in the regions corresponding to the main trabecular groups identified in literature, at minimal computational cost.

## Introduction

Bone tissue adaptation is a multiaspect physiological process driven by interrelated mechanical and biological stimuli (Zadpoor 2013) which requires the combined activity of osteoclasts and osteoblasts. It is thought that osteoblast activity is triggered by signals sent by the osteocytes (Burger and Klein-Nulend 1999; Temiyasathit and Jacobs 2010). Studies suggest that fluid motion in the extracellular space of the lacunar-canalicular porosities where the osteocytes lie may be involved in cellular mechanosensitivity (Rubin et al. 2001; Qin et al. 2003; Cowin et al. 1995; Temiyasathit and Jacobs 2010), potentially via the resulting shear stress on the cell walls due to fluid motion (Adachi et al. 2010). A potential candidate as an extracellular sensor of mechanical loading is the primary cilium, a microtubule that protrudes from the cell membrane (Whitfield 2003; Temiyasathit and Jacobs 2010).

In silico studies and simulations have implemented these theories in mechanistic models with probant results (Riddle and Donahue 2009; Adachi et al. 2010; Kameo and Adachi 2014; Pereira et al. 2015). Extensive work has also been conducted using phenomenological approaches, based on the empirical relationships between mechanical stimulus and bone adaptation (Huiskes et al. 1987; Adachi et al. 2001; Tsubota et al. 2002; Shefelbine et al. 2005; Scannell and Prendergast 2009; Phillips 2012; Marzban et al. 2013; Phillips et al. 2015; Geraldes et al. 2015). Such phenomenological approaches are limited in scale and scope, but present tremendous advantages in terms of model simplicity and computational efficiency.

**Fig. 1:**
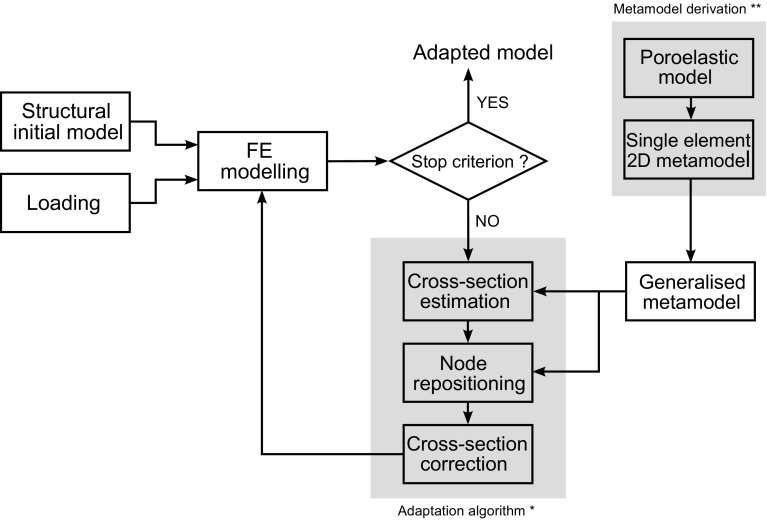
Work-flow used in this study. *Asterisk* modified from Phillips et al. (2015). *Doubleasterisk* derived in Villette and Phillips (2016)

In previous work, the authors developed a mesoscale structural modelling approach capable of predicting the internal structure of the femur based on the loading it was submitted to during daily activities (Phillips 2012; Phillips et al. 2015). In brief, cortical and trabecular bones were represented using shell and truss elements, respectively, and their thickness or cross-sectional area was iteratively adapted to reach a target strain under daily activity loading. The structural phenomenological modelling approach used in these studies presents limitations arising from the simplifications introduced in the truss formulation, where only axial strain is considered. In order to introduce a response to bending and shear, required for a complete description of the structural behaviour of bone, the authors considered the use of beam elements. This required a new formulation of the bone adaptation drivers.

In addition, it has been observed that bending-related loading scenarios lead to reorientation of the structure, aligning to the trajectory of the load (Adachi et al. 2001, 2010; Kameo and Adachi 2014; Tsubota et al. 2009). To account for the nodal repositioning involved in a structural model where trabecular struts are allowed to realign, the authors isolated phenomenological parameters with potential to drive this realignment (Villette and Phillips 2016). To this aim, they implemented and validated a representation of bone remodelling in a single trabecula treated as a microscale poroelastic continuum. This representation was used to implement a metamodel able to determine the change in cross section and end point position of a single beam representation of that same trabecula (Villette and Phillips 2016). Such a process is called ‘metamodelling’ or ‘surrogate modelling’. An inspiring example of metamodelling of bone structure was developed by Hambli (2011) who used a trained neural network to predict mesoscale remodelling based on macroscale FE computations. More recently, Kim et al. (2017) proposed and evaluated new macroscopic models for bone remodelling based on the microscopic mechanism of osteocytic mechanosensing to capture essential features from the complex microscopic mechanisms into a simple macroscopic model.

The aim of this study was to implement a phenomenological representation of functional adaptation of bone, modelled as a mesoscale lattice of beam elements, accounting for both element growth or abatement and reorientation. To this aim, the metamodel previously developed by the authors (Villette and Phillips 2016) was generalised to a lattice of beam elements and tested for the prediction of bone internal structure in a simplified structural model of the proximal femur.

## Methods

### Overview

A schematic of the work-flow used in this study is given in Fig. 1. An initial structural finite element (FE) model of the proximal femur was built with a randomised internal beam lattice structure and an outer shell layer based on the outer femur geometry extracted from computed tomography (CT). Five variations of a simplified load case representative of walking were simulated using *Abaqus*, and the strain results of these FE analyses were used to drive an iterative phenomenological adaptation (or remodelling) algorithm. This adaptation algorithm was modified from the authors’ previous work (Phillips et al. 2015) to include trabecular reorientation under loading in addition to the cross-sectional area adaptation (Villette and Phillips 2016). Based on the FE strain outputs, an initial estimation of adapted beam element cross-sectional areas is conducted, followed by the computation of updated beam nodal positions necessary for the beam reorientations. Based on the realised amount of element reorientation, a correction is made to the adapted cross-sectional area. The phenomenological rules controlling this algorithm were previously derived by the authors for a single trabecular element using a two-dimensional microscale poroelastic formulation of a continuous trabecula (Villette and Phillips 2016). In this study, these relationships were generalised into a strain-based metamodel for a three-dimensional lattice of trabecular structural beam elements.

### Finite element model and loading scenarios

#### Structural model

The femur structural mesh built in (Phillips et al. 2015) was modified for this study. In that project, a CT scan of a Sawbones fourth-generation composite femur (#3403) was processed in *Mimics* to create a volumetric mesh composed of 113,103 four-noded tetrahedral elements with an average edge length of 3.9 mm. The nodes and the element faces on the external surface were used to define three-noded linear triangular shell elements (*Abaqus* type S3), taken to be representative of cortical bone. Two-noded truss elements (*Abaqus* type T3D2) were defined between each node and the nearest sixteen neighbouring nodes. These were arbitrarily assigned a circular cross section with an initial radius of 0.1 mm. The resulting network was taken to be representative of trabecular bone.

**Fig. 2:**
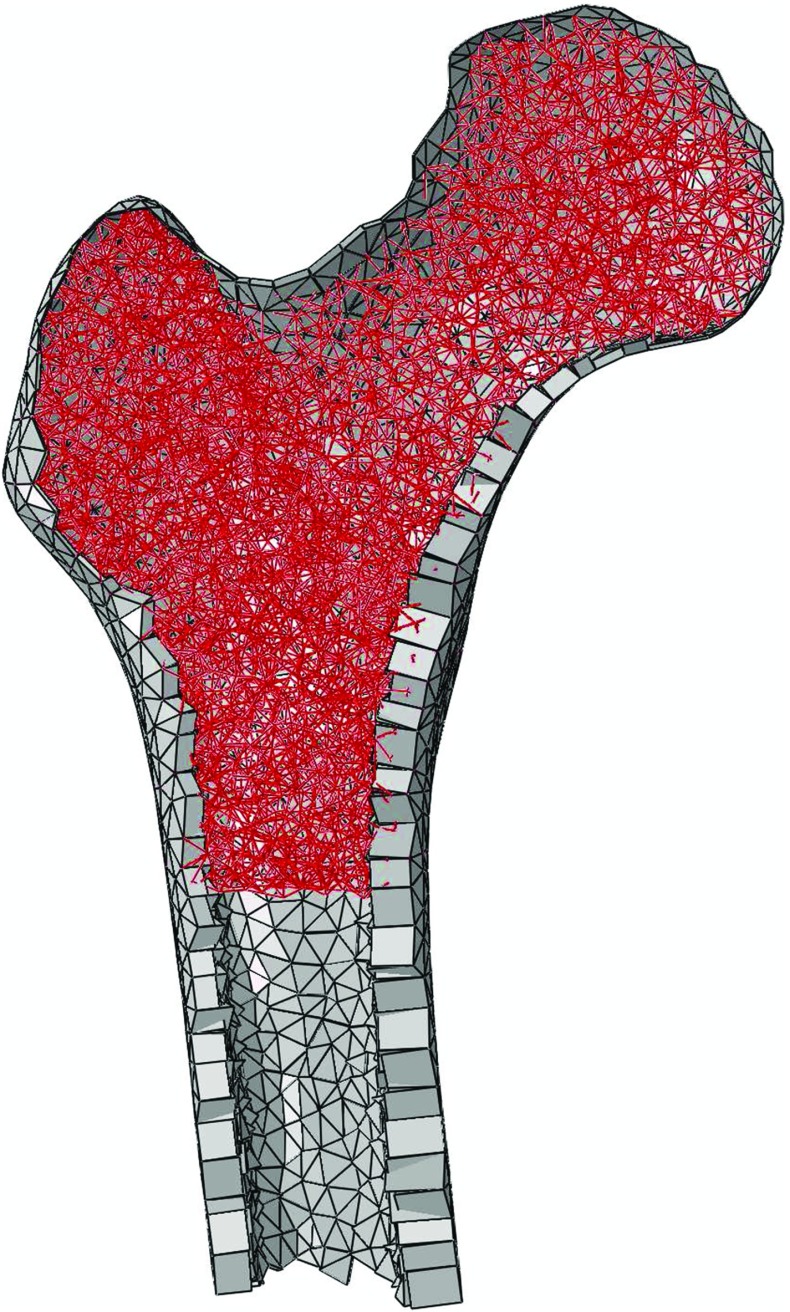
Cut of the initial proximal femur model with cortical and trabecular bone represented in *grey* and *red*, respectively

This structural model was simplified for this study. Only the proximal femur was considered, cut 85 mm distal to the lesser trochanter. The truss elements in the distal 50 mm of the newly cut model were also discarded. The nodal minimum connectivity within the trabecular bone was reduced from 16 to 6, keeping only the 6 shortest elements connected to each node. This reduction in number of elements was performed to increase the computational efficiency of the model, but also to ensure an easier assessment of the reorientation capability of the adaptation algorithm by reducing the number of initially available load paths, with the aim of encouraging some trabeculae to significantly reorientate. The truss elements were changed to quadratic Timoshenko beam elements (*Abaqus* type B32), keeping the truss start and end nodes and defining additional nodes at the mid point between these. All beams were assigned an initial radius of 0.1 mm. The shell elements were assigned the thickness value predicted in (Phillips et al. 2015) for a femur subjected to activities of walking, stair ascent and descent, sit-to-stand and stand-to-sit. A cut of the initial model is displayed in Fig. 2.

**Table 1:** Detail of the five loading cases applied on the proximal femur model

Position	X component (N)	Y component (N)	Z component (N)
Case 1*			
HIP 1	0	−2445	520
ABD 1	0	1175	−428
ITB	0	−625	0
Case 2			
HIP 2	−150	−2600	100
ABD 1	0	1175	−428
ITB	0	-625	0
Case 3			
HIP 3	0	−2445	520
ABD 1	0	1175	−428
ITB	0	−625	0
Case 4			
HIP 1	0	−2445	520
ABD 2	0	1175	−428
ITB	0	−625	0
Case 5			
HIP 1	0	−2445	520
ABD 1	0	1175	−428
ITB	0	−625	0
PSOAS	180	180	−90

‘*’ refers to the loading case used by Phillips (2012)

#### Loading

The loading applied was adapted from Phillips (2012), who used a simplified representation of loading experienced at the point of maximum hip joint contact force (HIP) associated with normal walking (Bergmann et al. 2001). That load case included a distributed load at the hip joint, as well as two point loads at the insertion of the iliotibial band (ITB) and the abductor muscles (ABD).

**Fig. 3:**
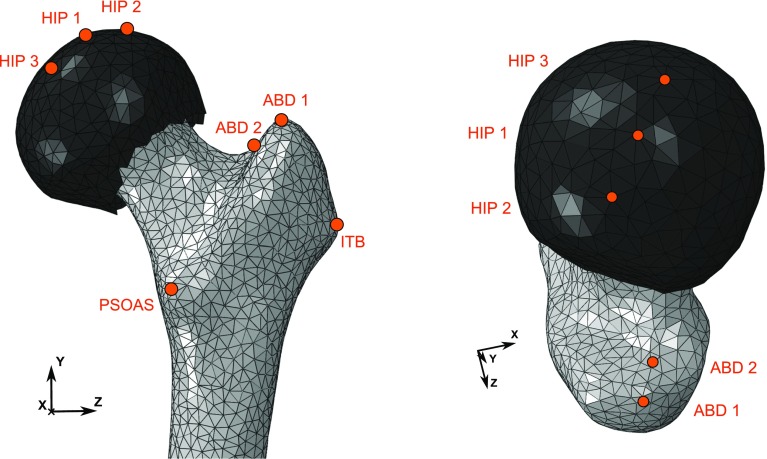
Position of the load application points on the proximal femur model

The authors have reported that an increased number of varied load cases yielded a more biofidelic structure (Phillips et al. 2015). In this study, four simplified load cases were chosen to complement that used by Phillips (2012). Two additional load cases involved a modified position of application for the hip contact force, and a third modified the position of application of the abductor muscle forces. The direction of application of the hip contact force was also modified. The last loading scenario involved an additional force exerted on the lesser trochanter, representative of the action of the Psoas muscle (PSOAS). These load cases were arbitrarily defined, based on observations made in previous work regarding the changes in direction of the hip joint contact force vector over a walking cycle, as well as the insertion point of the muscles exerting a significant force in the proximal femur during walking. They are detailed in Table 1, and the points of application are displayed in Fig. 3. The muscle loading was spread over the three closest nodes to the chosen insertion point. To ensure spreading of the hip contact force over a larger part of the surface area, a four-layer load applicator was built over the femoral head with six-noded linear continuum elements. The two inner layers were assigned a 2-mm thickness and cartilage-like material properties (*E* = 10 MPa, $\nu$ = 0.49). A parametric study (Villette 2016) was conducted on the thickness and Young’s modulus of the two outer layers to generate physiological surface stresses at the hip joint, using reports from in vitro tests (Brown and Shaw 1983) as reference. As a result, the top and second layers were made 3 mm and 2 mm thick, respectively. They were both assigned cartilage-like Poisson ratio ($\nu$ = 0.49) and respective Young’s moduli of 500 MPa and 10 MPa. The hip contact force was applied on one node on the outer layer of this applicator. All nodes on the distal boundary of the proximal femur model were fixed in translation and rotation.

**Fig. 4:**
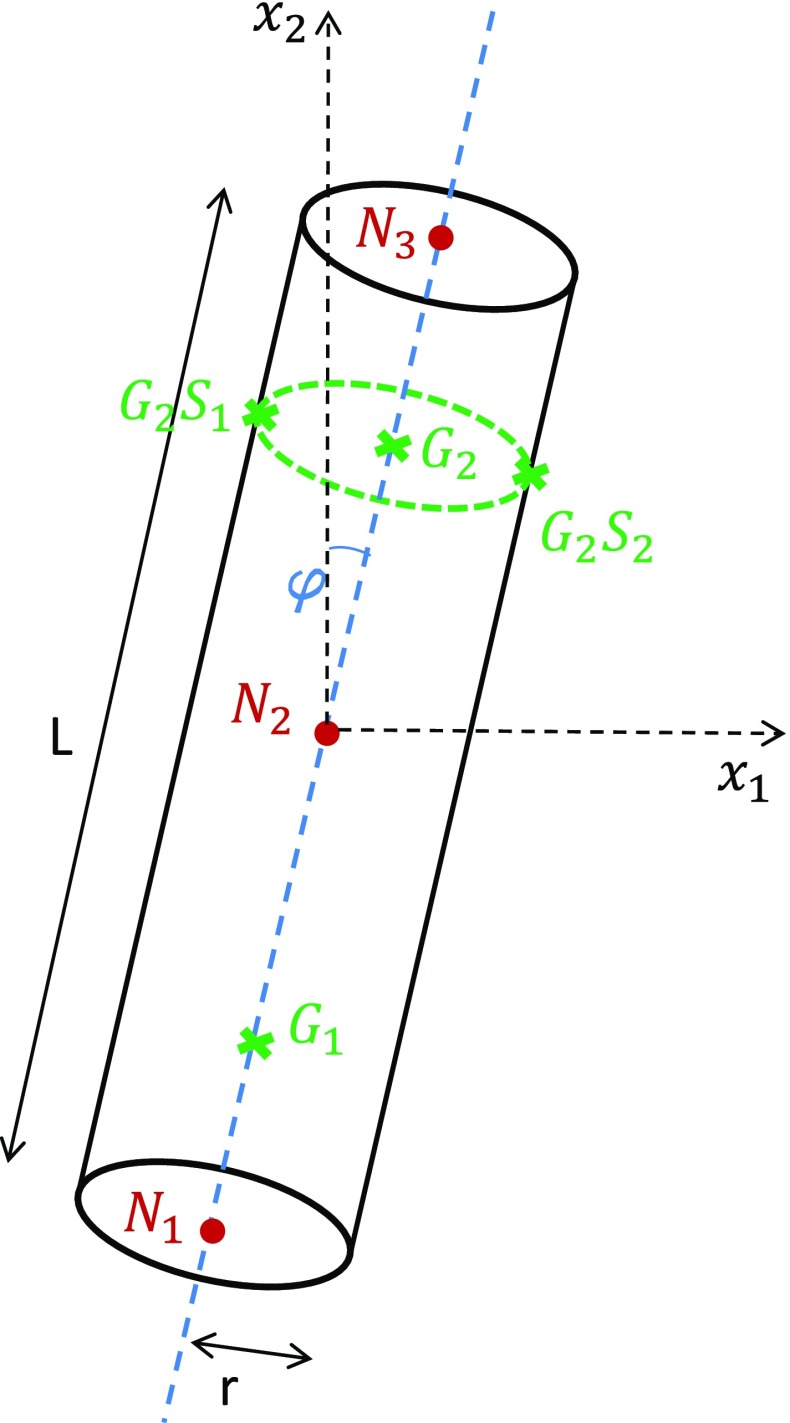
Schematic of the beam element parameters definition. (Reprinted from Villette and Phillips (2016) in accordance with the terms of the Creative Commons Attribution 4.0 International License)

### Iterative adaptation algorithm

#### Metamodel

In Villette and Phillips (2016), relationships were estimated which predict the change in cross-sectional area $R_\mathrm{A}$ and the angle of reorientation $\varDelta \varphi$ of a single trabecula modelled as a beam element in two dimensions, based on values of normal strain $\epsilon$ computed across the beam cross section at both integration (Gauss) points $G_1$ and $G_2$. The following notations were used, which are illustrated in Fig. 4. The indices *i* and *f* refer to the initial and final adapted states of the trabecula, respectively, and the notations $S_1$ and $S_2$ refer to the two opposite outer section points of the beam cross section in the plane of analysis.

$$\begin{aligned} \epsilon _{b}&=\epsilon _{G_2S_2}-\epsilon _{G_2S_1}\\ \epsilon _{a}&=\epsilon _{G_2}\\ K_{\epsilon }&=\dfrac{\epsilon _{b}}{\epsilon _{a}}\\ \varDelta \varphi&=\varphi _f-\varphi _i \\ R_\mathrm{A}&=\dfrac{A_f}{A_i} \end{aligned}$$

- $\varphi$: inclination of the beam with respect to the vertical axis
- $\varDelta \varphi$: change in beam inclination
- *A*: beam cross-sectional area
- $R_\mathrm{A}$: ratio of the beam initial and adapted cross-sectional areas
- $\epsilon _{b}$: relative difference in normal strain between diametrically opposite points on the outer surface of the beam cross-section. Also referred to as ‘bending strain’ in this study.
- $\epsilon _{a}$: normal strain at the beam central axis
- $K_{\epsilon }$: ratio of $\epsilon _{b}$ over $\epsilon _{a}$

The relationships predicting the change in cross-sectional area $R_\mathrm{A}$ and the angle of reorientation $\varDelta \varphi$ are defined in Eqs. 1 and 2, respectively.

1 $$\begin{aligned} R_\mathrm{A}=(iK_{\epsilon }+\text {sign}\,(\epsilon _{a})j)\epsilon _{a}+k \end{aligned}$$

with $$k=-0.065, i={-274.654}, j=999.7622$$

2 $$\begin{aligned} \varDelta \varphi =aK_{\epsilon }^{3}+bK_{\epsilon } \end{aligned}$$

with $$a=-0.1129, b=0.6725$$

#### Generalisation of the metamodel to a beam lattice in three dimensions

Equations 1 and 2 rely on a single computation of $K_{\epsilon }$ and $\varDelta {\varphi }$ for the whole element, under the understanding that this angle will be used to compute the displacements of both extremity nodes of the beam, with the same magnitude and opposite direction, which corresponds to a rotation of $\varDelta {\varphi }$ of the beam around its centre point.

**Fig. 5:**
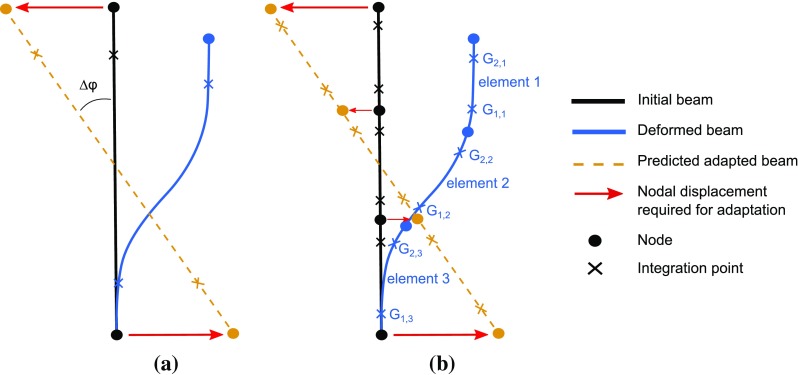
Schematic of a single beam and a series of three beams, submitted to the same downwards displacement to the right with *top node* fixed in rotation, in their initial, deformed and adapted shapes. *Note*: The deformed shapes shown are theoretical. The configuration shown in **b** includes enough elements to approximate this shape. In **a**, the single B32 beam element can predict translational and rotational nodal displacements consistent with this shape. However, the displacements of points located between the nodes, estimated by quadratic interpolation of the nodal variables, will not be consistent with this deformed shape

**Fig. 6:**
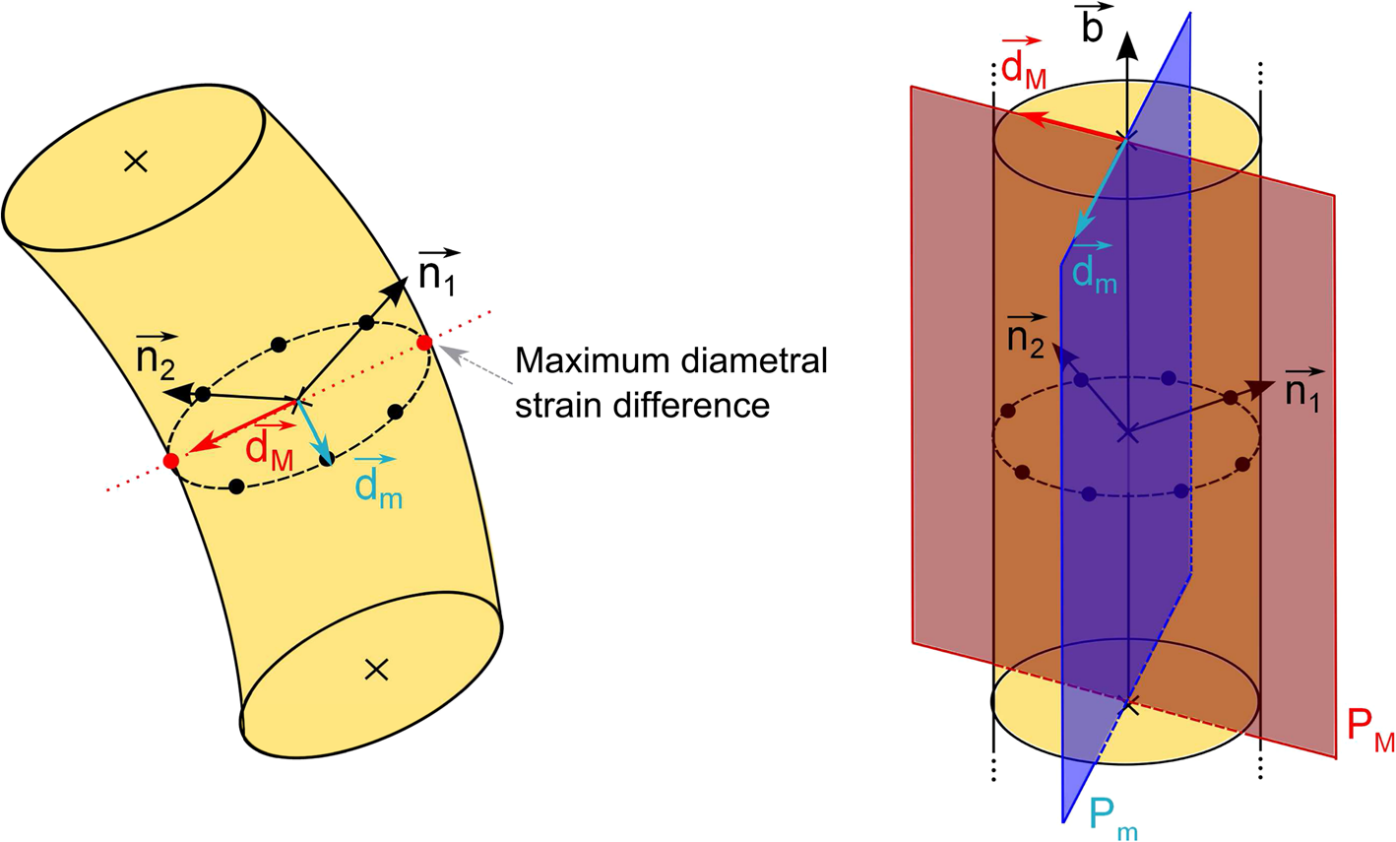
Determination of the beam bending planes at one integration point. Beam cross section in deformed state (*left*) and bending planes on the undeformed beam (*right*)

This mode of reorientation is relevant when considering a single beam in a strongly symmetrical loading scenario, as investigated in Villette and Phillips (2016). However, the current model includes interconnected chains of elements, which yields potentially important asymmetries in the deformation modes experienced by both extremities of a single beam. A clear example of such a situation is depicted in Fig. 5, where the deformation mode of a single bending beam is compared with the deformation of a chain of three beams under similar loading. To account for such phenomena, two computations of $\varDelta {\varphi }$, one per integration point, were conducted for each beam element, based on two separate computations of $K_{\epsilon }$. The values of $K_{\epsilon }$ and $\varDelta {\varphi }$ corresponding to integration point *i* in element *e* will be referred to as $K_{\epsilon ,e,i}$ and $\varDelta {\varphi }_{e,i}$, respectively. Associated displacements magnitudes $D_{e,i}$, in millimetres, were computed as follows:

3 $$\begin{aligned} D_{e,i}=\min \left( 0.1,0.5L\sin \,(\varDelta \varphi _{e,i})\right) \end{aligned}$$

where *L* is the initial beam element length.

In order to generalise the metamodel in three dimensions, a major bending plane had to be defined for each element, at both integration points. The three-dimensional beam elements in *Abaqus* allow for computation of variable fields at several section points with varying radial and angular position around each integration point. In this study, strain values were extracted at the centre as well as at 8 positions regularly distributed on the cross-sectional outer surface. Their position is defined as a function of the beam cross-section normals $\mathop {n_1}\limits ^{\rightarrow }$ and $\mathop {n_2}\limits ^{\rightarrow }$. The section points, beam normals and additional notations used in this section are displayed in Fig. 6.

At each integration point *i*, the plane of major bending $P_{M,i}$ was determined as the plane containing the unit beam direction vector $\mathop {b}\limits ^{\rightarrow }$ and the unit vector $\mathop {d_{M,e,i}}\limits ^{\rightarrow }$ joining the pair of diametrically opposed section points $G_iS_{1M}$ and $G_iS_{2M}$ presenting the highest absolute difference in normal strain (from the section point of lower index to that of higher index). A plane of minor bending $P_{m,i}$ was also defined, as the plane containing the unit beam direction vector $\mathop {b}\limits ^{\rightarrow }$ and the unit vector $\mathop {d_{m,e,i}}\limits ^{\rightarrow }$, perpendicular to $\mathop {d_{M,e,i}}\limits ^{\rightarrow }$ in the cross-section plane, and joining the pair of diametrically opposed section points $G_iS_{1m}$ and $G_iS_{2m}$.

To ensure accurate knowledge of the beam normal definitions, $\mathop {n_2}\limits ^{\rightarrow }$ was assigned to overwrite the automatic definitions computed by *Abaqus* which are not easily extracted. For each element, an initial $\mathop {n_{1,i}}\limits ^{\rightarrow }$ was set to [1, 0, 0], unless the angle formed between this vector and $\mathop {b}\limits ^{\rightarrow }$ was lower than 25$^{\circ }$. In that case, $\mathop {n_{1,i}}\limits ^{\rightarrow }$ was set to [0, 1, 0], unless the angle formed between this vector and $\mathop {b}\limits ^{\rightarrow }$ was also lower than 25$^{\circ }$. In this last case, $\mathop {n_{1,i}}\limits ^{\rightarrow }$ was set to [0, 0, 1]. With $\mathop {n_2}\limits ^{\rightarrow }$ defined as the cross product $\mathop {b}\limits ^{\rightarrow }\wedge \mathop {n_{1,i}}\limits ^{\rightarrow }$, and the final $\mathop {n_1}\limits ^{\rightarrow }$ defined as $\mathop {n_2}\limits ^{\rightarrow }\wedge \mathop {b}\limits ^{\rightarrow }$, the coordinates of the section points in the global coordinate frame could be precisely computed for each beam element in the undeformed configuration.

Based on these considerations, two ratios $KM_{\epsilon ,e,i}$ and $Km_{\epsilon ,e,i}$ were computed for each beam element *e*, for each integration point *i*:

$$\begin{aligned} {\textit{KM}}_{\epsilon ,e,i}&=\dfrac{\epsilon _{G_iS_{2M,e}}-\epsilon _{G_iS_{1M,e}}}{\epsilon _{G_{i,e}}}\\ {\textit{Km}}_{\epsilon ,e,i}&=\dfrac{\epsilon _{G_iS_{2m,e}}-\epsilon _{G_iS_{1m,e}}}{\epsilon _{G_{i,e}}} \end{aligned}$$

${{\textit{KM}}_{\epsilon ,e,i}}$ and ${{\textit{Km}}_{\epsilon ,e,i}}$ were limited to 1.4 in amplitude to restrain the use of the relationships to the domain where $\varDelta \varphi$ is monotonic (increasing).

#### First estimation of adapted cross-sectional area

Following each iteration *n*, an adapted cross-sectional area $A_{n+1}$ of each beam was computed as:

4 $$\begin{aligned} A_{n+1}=R_\mathrm{A}A_{n} \end{aligned}$$

with $R_\mathrm{A}$ computed based on Eq. 1. $K_{\epsilon }$ was taken as the value of maximum amplitude between $KM_{\epsilon ,e,1}$ and $KM_{\epsilon ,e,2}$. Finally, $\epsilon _a$ was taken as the normal strain of maximum amplitude between $\epsilon _{G_1}$ and $\epsilon _{G_2}$. For each beam element, the load case considered was that yielding the maximum $\epsilon _a$. Consistent with Villette and Phillips (2016), the beam cross-sectional area domain was linearly discretised into 99 categories between $\pi (0.1)^{2}~\hbox {mm}^2$ and $\pi (2)^{2}~\hbox {mm}^2$. The beam elements were assigned the closest cross-sectional area to the computed $A_{n+1}$ in this domain. A $100{\text {th}}$ category was added, which contained the elements whose adapted cross-sectional area fell under the arbitrary small area $0.001~\hbox {mm}^2$. These were set to a near-zero cross-sectional area of $\pi (0.001)^{2}~\hbox {mm}^2$.

#### Node repositioning

The nodal displacement $\mathop {D_{N}}\limits ^{\rightarrow }$ of all end nodes *N* was computed as a weighted average of the displacements associated with both major and minor bending modes in all the beam elements connected to them, as expressed below. For each beam element *e*, four values ${\varDelta \varphi }M_{e,1}$, ${\varDelta \varphi }M_{e,2}$, ${\varDelta \varphi }m_{e,1}$ and ${\varDelta \varphi }m_{e,2}$ were computed based on Eq. 2, with $K_{\epsilon }$ taken as $KM_{\epsilon ,e,1}$, $KM_{\epsilon ,e,2}$, $Km_{\epsilon ,e,1}$ and $Km_{\epsilon ,e,2}$, respectively. The associated displacements $DM_{e,1}$, $DM_{e,2}$, $Dm_{e,1}$ and $Dm_{e,2}$ were computed based on Eq. 3, with ${\varDelta \varphi }_{e,i}$ taken as ${\varDelta \varphi }M_{e,1}$, ${\varDelta \varphi }M_{e,2}$, ${\varDelta \varphi }m_{e,1}$ and ${\varDelta \varphi }m_{e,2}$, respectively. $S_N$ and $E_N$ are defined as the ensembles of beam elements connected to node *N* by their start or end node, respectively. The weighting coefficients were chosen as the index of the beam element cross-sectional area category $W_{A_{e}}$ when these are ranked in increasing order.

5 $$\begin{aligned} \begin{aligned} \mathop {D_{N}}\limits ^{\rightarrow }&= \dfrac{1}{\sum _{e\in {S_{N}}}W_{A_{e}}}\sum _{e\in {S_{N}}}W_{A_{e}}\left( DM_{e,1}\mathop {dM_{e,1}}\limits ^{\rightarrow }+Dm_{e,1}\mathop {dm_{e,1}}\limits ^{\rightarrow }\right) \\&\quad +\,\dfrac{1}{\sum _{e\in {E_{N}}}W_{A_{e}}}\sum _{e\in {E_{N}}}W_{A_{e}}\left( DM_{e,2}\mathop {dM_{e,2}}\limits ^{\rightarrow }+Dm_{e,2}\mathop {dm_{e,2}}\limits ^{\rightarrow }\right) \end{aligned} \end{aligned}$$

Updated node positions were constrained within the volume enclosed by the cortex. The positions of all beam middle nodes were updated as the middle point between start and end node updated positions.

Following the update of the node positions, the new beam normals $\mathop {n_2}\limits ^{\rightarrow }$ were computed following the process described in Sect. 2.3.2, and assigned to the elements for the next iteration. A copy of these normal definitions was stored in a text file to be retrieved when running the next iteration of orientation adaptation.

#### Cross-sectional area correction

In order to prevent excessive bone resorption observed in preliminary models, arising due to a difference in rates of cross-sectional adaptation and reorientation, the cross-sectional adaptation was conducted in two stages in each iteration: a preliminary adapted cross-sectional area $A_{n+1}$ was initially computed as described in 2.3.3 and set to its closest value in the modified domain. The orientation adaptation was then performed as described in 2.3.4. In the case when $A_{n+1}$ was found inferior $A_{n}$, the computation of a corrected cross-sectional area was then introduced at this point, to scale the cross-sectional adaptation based on the amount of reorientation effectively performed, to synchronise both adaptations. This step was not applied when $A_{n+1}$ was found superior to $A_{n}$.

To this aim, an estimate of the amount of effective reorientation $\varDelta \varphi _{\mathrm{eff},e}$ was computed for each beam *e* and compared to an estimate of the required reorientation ${\varDelta \varphi }M_{e}$. ${\varDelta \varphi }M_{e}$ was taken as the value of maximum amplitude between ${\varDelta \varphi }M_{e,1}$ and ${\varDelta \varphi }M_{e,2}$. The index of the corresponding integration point (‘1’ or ‘2’) was stored as $i_{\mathrm{max}}$. The plane of reorientation considered was taken as $P_{M,i_\mathrm{max}}$. $\varDelta \varphi _{\mathrm{eff},e}$ was computed as the angle between the newly reorientated beam direction in this iteration $n+1$ and the previous beam direction in iteration *n* in $P_{M,i_\mathrm{max}}$. A corrected cross-sectional area $Ac_{n+1}$ was then computed as follows for the elements presenting $A_{n+1}<A_{n}$:

6 $$\begin{aligned} Ac_{n+1}=A_{n}+\left( A_{n+1}-A_{n}\right) \min \left( \max \left( {\dfrac{\varDelta \varphi _{\mathrm{eff},e}}{{\varDelta \varphi }M_{e}}},0\right) ,1\right) \end{aligned}$$

#### Control adaptation algorithm

The functional adaptation algorithm was run over 50 iterations using the FE model and loading described in Sect. 2.2. In order to assess the changes generated by the implementation of the generalised metamodel compared to the original adaptation algorithm used by the authors (Phillips et al. 2015), a variation of this metamodel was run separately as control, using the same scenarios. Cross-sectional adaptation only was considered, with the influence of bending removed by setting $K_{\epsilon }$ to zero in Eq. 1. This is equivalent to adapting the cross-section of beam elements fixed in space based on a linear function of $\epsilon _{a}$. This adaptation is considered as virtually equivalent to the structural adaptation previously conducted by the authors (Phillips et al. 2015).

### Morphometry measurements

Bone volume density fields in the generalised metamodel were estimated in three dimensions using an in-house partition algorithm spanning the trabecular domain. In brief, each trabecular element was divided in segments of equal length to the spatial resolution characteristic of the partition. The volume of each segment was then added to the volume of the partition cell whose centroid was closest to the centre of that segment. Three iterations of convolution smoothing were used to attenuate the artefacts associated with using a discrete partition. Density measures are given as dimensionless values representing solid bone volume over total volume. Details of the partition resolutions used are provided in Results section.

The degree of anisotropy in the structure resulting from the generalised metamodel was quantified and compared to that of the control algorithm result. An in-house three-dimensional partition algorithm similar to that used to estimate density was used for these measures. In brief, the orientation of each trabecular element segment was stored as a unit vector in relation to the partition cell whose centroid was closest to the centre of that segment. These orientations were assigned a weighting related to the cross section of the corresponding trabecular element, and used to compute the weighted distribution of trabecular orientations within each cell. The results can be visualised for each cell in 2D polar plots, with angular coordinate representative of the orientation projected in the plane of interest and the radial coordinate representative of the prevalence (weighting) of that orientation in the partition cell. For clarity, the polar plots domains presented here are discretised in categories of orientations spanning 10 degrees, and the radial coordinates are normalised. The degree of anisotropy can also be visualised over entire slices of the model, by plotting the ‘major orientation’ of each partition cell. In this case, the ‘major orientation’ over a partition cell is representative of the most prevalent orientation in this cell. It is obtained using a K-Means (Lloyd’s) clustering algorithm (Lloyd 1982), and defined as the mean orientation of the largest of four clusters best defining the distribution of orientations.

**Fig. 7:**
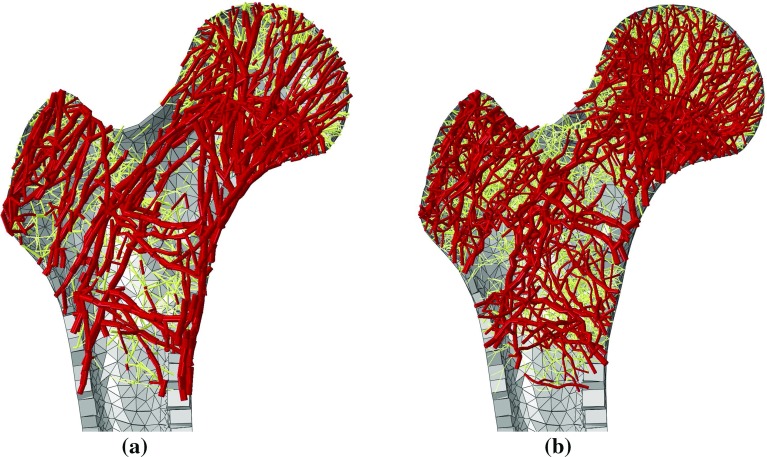
Adapted proximal femurs. Frontal posterior cuts of the cortex are displayed in *grey*. Trabecular elements across the full depth of the bone are displayed in *red* (radius $r\,>\,0.5$ mm ) and *light yellow* ($r\,\le \,0.5$ mm), with their cross-sectional area halved for clarity. The elements in the smallest category (near-zero radius) are not displayed (**a**) Generalised metamodel (**b**) Control algorithm

**Fig. 8:**
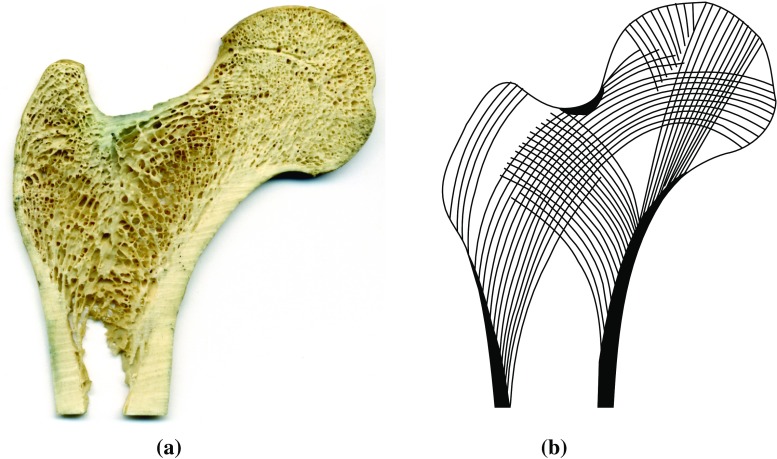
Clinical images of the human proximal femur **a** Photography of a longitudinal cut, **b** von Meyer (1867) anatomical drawing of the trabecular tracts (adapted by Phillips (2012))

## Results

The results of the two adaptations are presented in Fig. 7. Figure 8 displays clinical images of the proximal femur for comparison.

The metamodel adaptation resulted in a larger volume of trabecular bone ($39,500~\hbox {mm}^{3}$) compared to the control adaptation ($35,000~\hbox {mm}^{3}$). The number of trabecular elements reduced from 18,766 initially to 3864, and 10,873 at the end of the metamodel and the control adaptations, respectively. Consistent with these quantities, the trabecular lattice appears denser yet finer following the control adaptation in comparison with the metamodel.

**Fig. 9:**
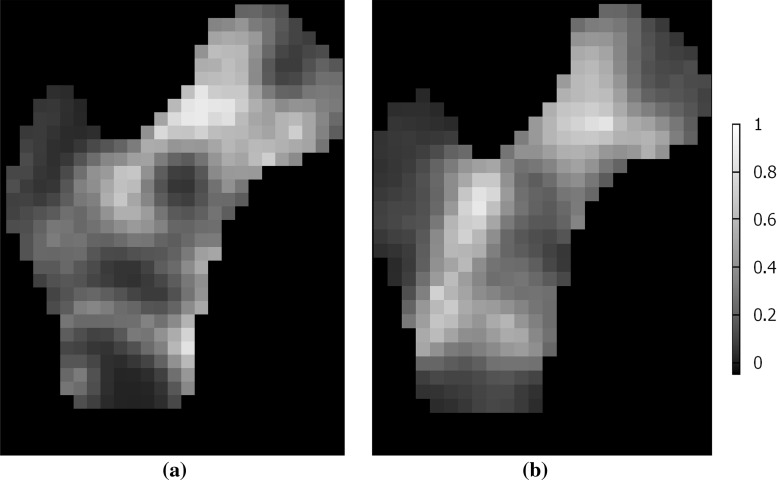
Density measures (volume of bone over surrounding total volume) in 10-mm-thick longitudinal slices of the trabecular structures, going through the centre of the femoral head with in-plane resolution of 4 mm. **a** Generalised metamodel, **b** Control algorithm

The main trabecular groups described in literature (Singh et al. 1970), as well as Ward’s triangle, can be observed clearly for the metamodel, and to a lesser extent in the control. Characteristic structural features of the human proximal femur (Vahdati et al. 2014; Fyhrie and Carter 1990) including dense cross-shaped area in the centre of the head where primary compressive and tensile trabecular groups meet and sparser neck and greater trochanter are also visible in the density plots shown in Fig. 9. When multiplied by solid bone volumetric mass density of $2~\hbox {g/cm}^{3}$ (Keaveny et al. 2003), the density distributions calculated in both models compare well with measures reported in literature (Fyhrie and Carter 1990; Yang et al. 2012), ranging from around $0.2-0.3~\hbox {g/cm}^{3}$ in the neck and the greater trochanter, to close to solid bone ($1.8~\hbox {g/cm}^{3}$) in some areas of the head. These density plots also illustrate the high heterogeneity of the structures generated, with some important structural features including part of the primary compressive and greater trochanter groups not being picked up in the chosen longitudinal slice. This is particularly visible in the metamodel structure, which also presents a clearer Ward’s triangle and a sparser distal region where the proximal femur transitions into the shaft. It should be noted that the density plots only consider trabecular bone, whose domain is larger in the metamodel than in the control due to the elements repositioning. For this reason, the proximal femur contours on both density plots appear slightly different, although the same femur outer shape was used for both models.

**Fig. 10:**
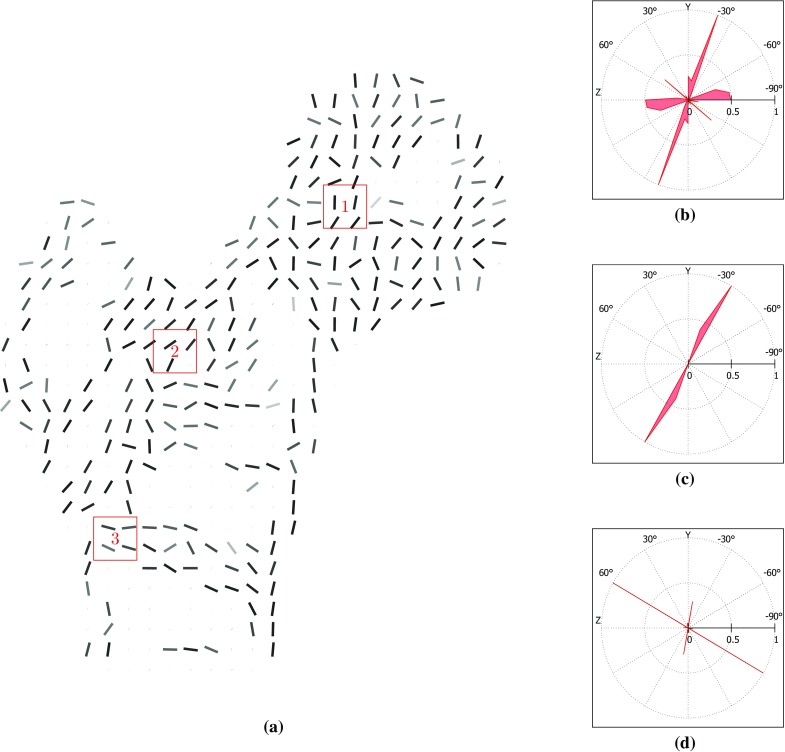
Measures of the degree of anisotropy in the generalised metamodel trabecular structure. **a** Major trabecular orientations in the longitudinal 10-mm-thick slice going through the centre of the head with in-plane resolution of 4 mm. *Grey level* is indicative of the in-plane component of this orientation (*dark shades* indicate close-to-in-plane orientations). Space is left empty when not enough trabecular material is present to compute orientations. (**b**,**c**,**d**) Normalised weighted distribution of trabecular orientations within 10-mm large cubic partition cells defined in (**a**). Angular coordinates are representative of orientations and radial coordinates are representative of their prevalence. The orientations are projected in the (YZ) plane

**Fig. 11:**
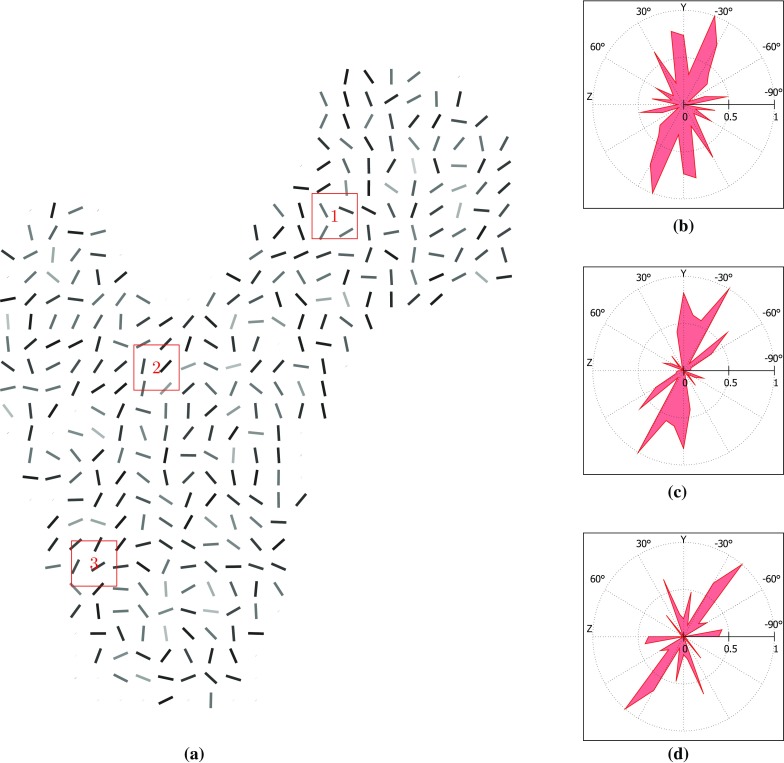
Measures of the degree of anisotropy in the control algorithm structure. **a** Major trabecular orientations in the longitudinal 10-mm-thick slice going through the centre of the head with in-plane resolution of 4 mm. *Grey level* is indicative of the in-plane component of this orientation (*dark shades* indicate close-to-in-plane orientations). Space is left empty when not enough trabecular material is present to compute orientations. (**b**,**c**,**d**) Normalised weighted distribution of trabecular orientations within 10 mm large cubic partition cells defined in (a). Angular coordinates are representative of orientations, and radial coordinates are representative of their prevalence. The orientations are projected in the (YZ) plane

Figs. 10 and 11 illustrate the degree of anisotropy in the generalised metamodel and control trabecular structures. In the generalised metamodel structure, the major trabecular orientation shows a good correlation with the main trabecular group orientations displayed in Fig. 8b and reported in literature (von Meyer 1867; Enns-Bray et al. 2014; Kersh et al. 2013). This is particularly true for the primary compressive group, the greater trochanter group and the thin secondary compressive group. It is also highly visible for the part of the primary compressive group which appears on this slice. In the control model, the alignment of the major trabecular orientations with clinical observations is less evident. Some consistency is observed in the primary tensile group and in the primary and secondary compressive groups. However, significant variations in major orientation are observed between consecutive cells, which does not allow the definition of smooth trabecular trajectories spanning several centimetres. Consistent with these observations, the distributions of trabecular orientations at selected locations show a small number of well-defined trabecular orientations spanning the areas in the generalised metamodel while trabecular orientations are more spread out in the control model.

## Discussion

The overall directionality of the trabecular groups is better defined on the structure resulting from the metamodel than on the control adaptation structure. The visibly smoother trabecular lines shown in Figs. 7a and 10 compared to Figs. 7b and 11 illustrate the reorientation capabilities of the metamodel. In addition, the major trabecular orientations of the metamodel are more consistent with observations on native femur slices or clinical images reported in the literature (von Meyer 1867; Koch 1917; Enns-Bray et al. 2014; Kersh et al. 2013) than the major orientations of the control. Furthermore, the main orientations measured in location 1 in the metamodel are consistent with the intersection of the primary compressive and tensile groups as depicted in Fig. 8b. Similarly, the main orientations measured in location 3 in the metamodel are consistent with the intersection of the primary tensile and secondary compressive groups. The main orientations measured in location 2 suggest a strong prevalence of elements aligned with the primary tensile group, as would be expected from clinical observations. It should be noted that weightings have been used to measure degrees of anisotropy in this study. For this reason, a small number of thin elements with non-prevalent orientations can exist which do not impact these measures and will not be visible on the polar plots if their own weighting is negligible. Rough alignment of the trabecular orientations with the primary tensile group tracts is clearly visible from the polar plots at all three locations in the control model. However, the existence of clear other trabecular trajectories is negated by the high number of trabecular orientations of similar importance measured, which is inconsistent with clinical observations (von Meyer 1867; Koch 1917). In conclusion, the metamodel results in better alignments of its main trabecular orientations with the proximal femur trabecular tracts described in literature (von Meyer 1867; Koch 1917; Enns-Bray et al. 2014; Kersh et al. 2013) which strongly highlights the improvement brought by the metamodel to the accuracy of bone structure representation.

From qualitative assessment of the metamodel structure in Figs. 7a, and quantitative measures in Fig. 10b and 10d, it appears that the intersections of trabecular tracts are not orthogonal. In his drawings, von Meyer (1867) did not report them as orthogonal. However, Wolff (1869) later admonished him for what he considered as an omission. To this day, the debate between supporters (Koch 1917; Pauwels 1950; Hayes and Snyder 1981) and critics (Zschokke 1892; Carter et al. 1989; Skedros and Baucom 2007) of Wolff’s trajectorial theory is not settled. Although conducted at low resolution with simplified loading scenarios, the present study is more in line with the latter. Several authors have suggested that the non-orthogonal intersections of trabeculae in the human proximal femur may represent a more optimal design for resisting shear stresses (Skedros and Baucom 2007; Pidaparti and Turner 1997), presumably more prevalent in the human femoral neck than in other bones such as the calcanei of deer or sheep where orthogonal intersections of trabecular tracts have been observed (Lanyon 1974).

Both adapted structures present numerous thick elements, with a radius close to the upper limit, in localised areas. This is due in part to the chosen scale and the low initial connectivity, which limited the initial number of elements, and thus the opportunities to spread the load, yielding high load transfer through the elements localised near the points of load application. In addition, only a very small subset of loading scenarios representative of daily activity loading was applied to these models; a broader range of load cases would act against excessive specific specialisation of the structure (Phillips et al. 2015; Villette 2016). Future work should consider investigating a reorientation adaptation based on the average of the reorientations predicted for each individual load case, rather than considering only the load case responsible for the highest strain on the beam central axis as was done here.

Realignment of trabecular elements is supposed to reduce bending in the structure and thus increase the structural efficiency of the modelled bone architecture. For this reason, the higher bone volume resulting from the metamodel adaptation compared to the control adaptation is unexpected. However, the control algorithm only takes into account the normal strain measure on the central axis of the beam; high surface strains arising from bending are thus not considered when driving the adaptation, which partly explains the lower required bone volume compared to the metamodel. In order to clarify this point, a modified version of the control algorithm was run, driven by the normal strain of maximum amplitude over the whole beam cross section. The resulting trabecular volume amounted to over $110,000~\hbox {mm}^{3}$, close to three times the trabecular bone volume required in the metamodel. This observation supports the argument in favour of a higher structural efficiency of the metamodel over the control model. In addition, it should be noted that a trabecular group is forming in the medial cortex region beneath the femoral head in the metamodel adaptation, which is not observed in the control adaptation. Trabecular beam elements growing in the medial cortex region amount to about $4000~\hbox {mm}^{3}$ in the metamodel adaptation, compared to only $800~\hbox {mm}^{3}$ in the control adaptation. This phenomenon accounts for over half of the difference in trabecular bone volume between the two adaptations.

The metamodel adaptation algorithm presented in this study does not support beam elements redefinition. For this reason, new connections between elements, or merging of superposed elements is not allowed, and neither are bifurcations or suppression of load paths. Further developments of the algorithm will focus on implementing these capabilities.

It is thought that growth of trabecular beam elements in the medial cortex region originates from the particular cortical representation used in the authors’ models. The shell elements used to represent bone cortex present a reduced number of nodes, only present on the outer femoral surface. For this reason, load transfer between beam and shell elements is limited to a reduced number of points on the outer surface. Alignment of interconnected beam elements overlapping with the shell thickness is likely to increase the efficiency of the load transfer mechanism in this region. Future versions of the structural models may benefit from a more comprehensive representation of the transition between cortical and trabecular bones. For example, use of continuum shell elements, with twice as many defining nodes as the conventional shell elements, could be considered.

The number of 50 iterations used here was arbitrarily set, although the stabilisation of the structural adaptation in the preceding iterations was qualitatively checked. Further work should focus on the implementation of a quantitative convergence criterion to control the number of iterations required.

The quadratic beam model of the proximal femur model used here counts 18,766 beam elements and a total of 150,000 variables. Its CPU time to run load Case 1 is 20 s. This is a significant increase in computational efficiency when compared to a purely microscale poroelastic model used to derive the relationships defining the metamodel (Villette and Phillips 2016), which requires around 9 s to run a simple load case on a single trabeculae, equivalent to one single beam, which would correspond to around 47  h for a full proximal femur model. Based on these considerations, the metamodel allows for an increase in computational efficiency of around four orders of magnitude. However, it should be noted that the use of quadratic beams, required to use the metamodel, has a cost in terms of computational efficiency compared to the truss models previously used by the authors (Phillips et al. 2015). Indeed, the truss model equivalent to the proximal femur model used here counts only 30,000 variables, and runs in 7 s.

## Conclusion

A metamodel developed based on two-dimensional microscale poroelastic remodelling analyses (Villette and Phillips 2016) was generalised to a three-dimensional lattice of multiple trabecular elements. It was applied to a simple structural model of a proximal femur made of around 19,000 elements, submitted to a simplified set of loading cases, and was able to capture realignment of trabecular elements consistent with the main trabecular groups observed in the native femur. With a CPU time of 20 s to run a simple load case, this model has strong potential for an effective compromise between accuracy of bone structure representation and computational efficiency. The main limitation of the bone remodelling metamodel at this stage is the lack of definition and implementation of a convergence criterion, which should be prioritised in future work.

Future work will include the adaptation of long bones made of a finer mesh, submitted to more representative load cases, for increased resolution, and better assessment of the capabilities of the metamodel when compared to the purely phenomenological models (Phillips 2012; Phillips et al. 2015; Geraldes and Phillips 2015).

In addition to the improvement they can bring to bone remodelling predictions at meso- to macroscales incompatible with mechanistic models of cellular biology and biochemistry, the poroelastic model and the derived metamodel present potential for use within multiscale and multiphysics approaches, typically where living cells would be considered. For instance, it makes it possible to consider a combined model with poroelastic regions where localised cell mechanical stimulus is of interest, while conserving computational efficiency in the majority of the volume of the model. The metamodel could also be adapted to take into account alteration of cellular mechanotransduction, such as a reduced threshold for stimuli sensing, or a modified response to stimuli, with applications in osteoporosis and osteoarthritis investigation.
